# West Nile virus outbreak in humans and epidemiological surveillance, west Andalusia, Spain, 2016

**DOI:** 10.2807/1560-7917.ES.2018.23.14.17-00261

**Published:** 2018-04-05

**Authors:** Nuria López-Ruiz, María del Carmen Montaño-Remacha, Enric Durán-Pla, Mercedes Pérez-Ruiz, Jose María Navarro-Marí, Celia Salamanca-Rivera, Blanca Miranda, Salvador Oyonarte-Gómez, Josefa Ruiz-Fernández

**Affiliations:** 1 Surveillance Department, Andalusian Regional Ministry of Health, Seville, Spain; 2 Department of Preventive Medicine and Public Health, University Hospital Puerta del Mar, Cadiz, Spain; 3 Department of Microbiology, University Hospital Virgen de las Nieves, Granada, Spain; 4 Institute of Biosanitary Research, Granada, Spain; 5 Network Cooperative Research in Tropical Diseases (RICET), Carlos III Institute of Health (ISCIII), Madrid, Spain; 6 Department of Preventive Medicine and Public Health, University Hospital Virgen del Rocío, Seville, Spain; 7 Transfusion Tissues and Cells Center, Seville, Spain; 8 General Secretary for Public Health and Consumption, Regional Ministry of Health, Andalusia, Spain

**Keywords:** West Nile fever, infection control, outbreaks, surveillance, laboratory surveillance, epidemiology, Andalusia, vector-borne disease, viral infections

## Abstract

In Andalusia, Spain, West Nile virus (WNV) surveillance takes place from April to November, during the active vector period. Within this area seroconversion to this virus was evidenced in wild birds in 2004, affecting horses and two humans for the first time in 2010. Since 2010, the virus has been isolated every year in horses, and national and regional surveillance plans have been updated with the epidemiological changes found. WNV is spreading rapidly throughout southern Europe and has caused outbreaks in humans. Here we describe the second WNV outbreak in humans in Andalusia, with three confirmed cases, which occurred between August and September 2016, and the measures carried out to control it. Surveillance during the transmission season is essential to monitor and ensure prompt identification of any outbreaks.

## Introduction

West Nile Virus (WNV) is a virus of the *Flaviviridae* family. Birds are the primary reservoir hosts while mosquitoes are the vectors, following a bird-mosquito enzootic cycle [1]. Humans and horses are accidental dead-end hosts, not contributing to the spread of the disease. WNV is most commonly transmitted to humans by mosquito bites (genus *Culex*) and rarely through other routes such as organ transplantation, blood transfusion, from mother to child during pregnancy, delivery or breastfeeding, and through mishandling of samples in the laboratory [2]. In west Andalusia, the most frequent species associated with WNV transmission are *Culex pipiens* and *Culex theileri* [3]. Most of the infections in humans (80%) are asymptomatic and less than 1% of infected cases develop a severe disease such as neuroinvasive disease, myocarditis, pancreatitis or fulminant hepatitis [4]. In humans, the peak of viraemia is 4–8 days post-infection and anti-WNV IgM appears when viraemia is resolved and symptoms arise. The incubation period is between 2 and 14 days [1]. Treatment of the infection is supportive. There is no vaccine for humans and there are no specific antiviral drugs.

WNV was first isolated in 1937 in the West Nile district of Uganda, and from the 1950s to the 1980s, it was found in mosquitoes, birds, and mammals in different countries of Europe, Africa, Australia, and Asia, with sporadic symptomatic cases in humans [5]. However, since the beginning of the 21st century, this disease has emerged in the form of outbreaks and epidemics, with a significant proportion of cases occurring in Europe and North America [2,6,7], posing a threat to public, human and animal health.

There are two main WNV genetic lineages. Lineage 1 is responsible for the majority of the outbreaks in horses and humans in Europe, Africa, the Middle East, India, Australia, and North America. The WNV lineage 2 (WNV2) is believed to have entered Europe 2 years before the first isolation in Hungary in 2004. After this, around 2007, WNV2 spread west towards Austria and east towards Greece with suggestions of a period of enzootic circulation in Europe involving reservoirs and vectors before human involvement. Both lineages have similar pathogenicity characteristics in humans [2,8-10].

In Spain, WNV circulation in birds was confirmed in 2004 [11,12], and only one human case was retrospectively diagnosed in that year [5,13]. In September 2010, the Spanish Ministry of the Environment, and Rural and Marine Affairs notified the detection of WNV in several horses in three provinces of Andalusia (Seville, Huelva and Cádiz), a southern territory with a large horse-farming tradition. In this year, there were two confirmed human cases, considered to be the first outbreak in the area. The surveillance during the active vector period, from April to November, from 2011 to 2015, also detected virus activity in horses and wild birds.

According to the International Health Regulations (2005), notification of WNV in humans is mandatory as it constitutes ‘an unusual or unexpected event that may have serious public health repercussions and can have a quick international spread’ [14]. For the European Surveillance Network (Early Warning and Response System, EWRS) notification became mandatory in December 2007 and in the same year Spain created its first national WNV monitoring plan [15]. Since 2010, according to Andalusian WNV protocol, human surveillance and preventive measures start every April, at the beginning of the active vector period [5].

### The event

On 11 August 2016, the French National Reference Centre for Arboviruses (NRC, Institut de Recherche Biomédicale des Armées, Marseille) confirmed a case of West Nile Neuroinvasive Disease (WNND) in a French man in his mid-70s returning from Andalusia, Spain, after spending 43 days between Seville and Huelva provinces (from 22 June to 4 August 2016). The patient reported fatigue and chills on 26–27 July, and on 29 July he consulted a physician for fever and sore throat. On 4 August, he and his family decided to return to France. When he arrived in France, his condition deteriorated, and he was admitted to the intensive care unit of a hospital, where he was diagnosed with meningoencephalitis. Serological assays in a cerebrospinal fluid (CSF) sample collected on 8 August detected anti-WNV IgM and IgG. His evolution was benign without sequelae. The Spanish division in the EWRS network alerted Andalusian Health Authorities of the confirmation of this first case. After this notification, surveillance and implemented measures were heightened in the region. In September 2016, two additional cases were found at local level.

Here, we present the results of this outbreak investigation, the measures in response to the human cases and the human surveillance system plus a brief description of animal and vector surveillance involved in this WNV outbreak in west Andalusia 2016.

## Methods

### Human surveillance system in Andalusia

In Andalusia, the statutory regulation of 19 December 1996 (updated on 12 November 2015), contains a list of mandatory notifiable diseases, including WNV. This is as an emerging disease and a single case leads to a public health alert, which requires urgent notification to the Epidemiology Andalusian Surveillance System (SVEA) [16]. Its purpose is to collect information from new cases, to define strategies of infection prevention and control, and to establish measures for contacts and/or the environment. It is based on communication between local and regional authorities in under 24 hours, through designated epidemiologists responsible for each healthcare district at both primary and secondary level.

The SVEA WNV protocol contains all the information and strategies to control and prevent WNV infections. The case definition used is European Union 2012 (EU 2012) with two more clinical inclusion criteria: Guillain–Barré syndrome and acute flaccid paralysis [5]. Cases are sub-classified as probable, confirmed or imported, following the European criteria [17]. A zone is considered a risk area after detection of confirmed cases in horses and/or humans.

The WNV surveillance in humans is conducted by SVEA in Andalusia since 2010. The protocol contains instructions to be carried out in the active vector period, from April to the end of November. The surveillance in humans is based on active case finding in patients with a suggestive clinical presentation and no other aetiology identified. This active surveillance is heightened after a confirmed animal or vector case. Blood donation controls are not routinely performed unless human cases are identified.

The reference laboratory for human WNV infections in Andalusia is the Virgen de las Nieves Hospital, Granada. Tests were performed (serological and/or molecular assays) with serum, urine and/or CSF samples from probable cases. The presence of WNV antibodies and WNV RNA were investigated with the WNV IgM Capture DxSelect kit (Focus Diagnostics, Cypress, California, USA) in serum and CSF and a real-time (RT)-PCR in serum, CSF and urine, respectively [18]. The WNV NAT (Techniques of Nuclear Acid Detection) screening in blood donations was performed in individual samples, as required by Directive 2014/110/EU of 17 December 2014 [19], using a WNV assay from the Procleix Panther system (Grifols, Barcelona, Spain).

### Animal and vector surveillance system in Andalusia

Since 2003, the surveillance of WNV in animals has been conducted by the Agriculture Service in Andalusia in partnership with the Veterinary Service of the Ministry of Agriculture and Fisheries Food and Environment (MAPAMA). Horse and bird (migrant and non-migrant) surveillance is based on active, passive and sentinel detection. It runs from July to November [20]. The reference animal health laboratory of the National Institute of Agricultural and Food Research and Technology (INIA), is located in Algete (Madrid), and is part of the Central Veterinary Laboratory. The diagnostic methods are similar to human cases and the case definition is focused on laboratory results [21]. Routine vaccination is not performed for horses. Additionally, there is mosquito surveillance involving species identification and the presence of WNV, every year, from April to December [22].

### Data collection and descriptive analysis

Information regarding the demographic characteristics, clinical manifestations and laboratory results of the human cases diagnosed in west Andalusia in 2016 were collected through the SVEA WNV questionnaire [5]. The time frame and geographic location of the infection and a detailed clinical description of the human cases were obtained. EWRS provided information on the first case diagnosed in France. In addition, serological outcomes of infected horses in west Andalusia were collected. Also, data regarding blood donation such as number of donations and serological outcomes in the risk areas were retrieved.

## Results

### Human West Nile virus cases in 2016

After the notification of the first case, intensified human surveillance detected two further human cases. The second case was confirmed on 1 September in a man in his early 70s residing in a village in Seville province. He was admitted to the nephrology service after 5 days with emetic and diarrhoeal syndromes. Subsequently, he presented dysarthria, momentary loss of vision and right hemiparesis. WNV meningoencephalitis was confirmed by the detection of anti-WNV IgM in the CSF and serum samples. RT-PCR from the CSF, serum and urine samples tested negative. His evolution was benign without sequelae.

On 8 September, the third case was confirmed in a man in his early 50s, who had received a liver transplant years before, and who was also resident in a village in Seville province. This patient attended accident and emergency with a 5-day fever, and was hospitalised. A lumbar puncture was performed due to the presence of signs and symptoms of meningoencephalitis. Anti-WNV IgM was detected in the CSF and 11 days later, in the serum samples. Low-level viruria was assessed by a Ct (crossing threshold cycle) value of 38 and 39 in the real-time reverse transcriptase (RT)-PCR from two replicates of the urine sample. Generic RT-PCR [21] was carried out for sequencing purposes to determine the WNV lineage, which did not yield any results. His evolution was benign, with cognitive disorders and sequelae in the peripheral facial nerve.

The summary of the cases is shown in Table 1.

**Table 1:** Demographic, epidemiological and clinical data of cases of West Nile virus infection, west Andalusia, Spain, 2016 (n = 3)

	Case 1	Case 2	Case 3
**Date of first symptoms (2016)**	**26 July**	**16 August**	**14 August**
**Notification date**	11 August	8 September	1 September
**Age (years)**	Mid-70s	Early 50s	Early 70s
**Sex**	Male	Male	Male
**Province**	Seville Huelva	Seville	Seville
**Symptoms**	Fatigue Chills Fever (> 38.5 °C) Somnolence Weakness	Fatigue Fever (> 38.5 °C) Somnolence	Emetic diarrhoeal Vomiting Fever (> 38.5 °C) Momentary loss of vision Dysarthria
**WNV clinical presentation**	Meningoencephalitis	Meningoencephalitis	Meningoencephalitis
**Serological test** **(CSF)**	IgM pos IgG pos	IgM pos	IgM pos
**RT-PCR**	NA	Urine pos	Negative CSF Negative serum sample Negative urine
**Comorbidity**	NA	DM II Cholelithiasis Liver transplant	CKD V HBP Klinefelter syndrome Thyroidectomy
**Evolution**	Benign	Cognitive disorders Facial nerve	Benign

CKD V: chronic kidney disease V; CSF: cerebrospinal fluid; DM II: diabetes mellitus type II; HBP: high blood pressure; NA: not available; pos: positive.

### Public health measures

In April 2016, according to SVEA WNV protocol, human surveillance began in Andalusia [5]. In the areas where there was circulation of the virus during the previous season, measures were activated at the beginning of the season. These were: to provide information to the population with an intensive press campaign about how to avoid mosquito bites (individual measures); cleaning potential mosquito-breeding sites; recommendations for tissue-sample handling; post-mortem examination and safety in transfusions and transplants.

After the first human case, a multidisciplinary work group was created. This team coordinated communication between agriculture, environmental and public health departments. A new measure was activated, control of the blood donation system, according to SVEA WNV protocol and EU and Spain regulations in risk areas [5,23]. All donations in the affected areas were blocked during the incubation period (according to the first case), to perform the WNV NAT screening in stored plasma samples. Also, a retrospective NAT screening was conducted in all donations that took place in the affected areas from 26 July. In addition, blood collections were cancelled in high-risk areas (radius of 10 km from districts visited by case 1 during his incubation period) and were re-established on 18 August 2016, following the introduction of WNV NAT screening.

Active search for cases (all unexplained meningitis and meningoencephalitis from areas with affected horses) started at the beginning of the season and was intensified after the first case, identifying 30 probable cases, of which two were confirmed (Table 2).

**Table 2:** Screening of unexplained meningitis and meningoencephalitis cases by province, west Andalusia, Spain, 2016^a^ (n = 30)

Province	Unexplained cases of meningitis and meningoencephalitis (n)	Confirmed cases of WNV infection (n)
**Cádiz**	6	0
**Seville**	17	2
**Málaga**	3	0
**Huelva**	4	0
**Total**	30	2

WNV: West Nile virus.

^a^ During the WNV alert, from 26 July to 30 November 2016.

**Figure 1:**
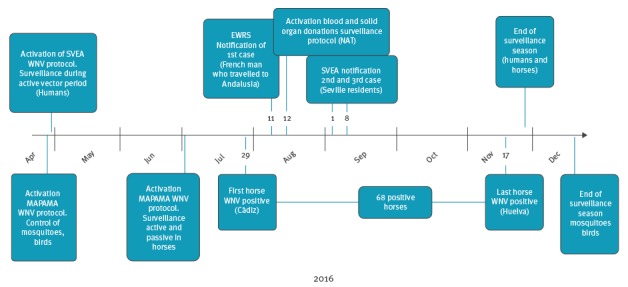
Timeline of West Nile virus infection outbreak, west Andalusia, Spain, 2016

EWRS: Early Warning and Response System; MAPAMA: Ministry of Agriculture and Fisheries Food and Environment; NAT: Techniques of Nuclear Acid Detection; SVEA: Epidemiology Andalusian Surveillance System; WNV: West Nile virus.

In Andalusia, there are fixed and unfixed points for blood donations. An intensive monitoring of donations was performed from 26 July to 30 November 2016. A total of 9,457 donations belonging to the affected areas or at risk of WNV infection were studied; 852 corresponded to stored plasma samples. No positive results were found. A summary of the outbreak timeline and public health measures is shown in Figure 1.

### Animals and vector surveillance

**Figure 2:**
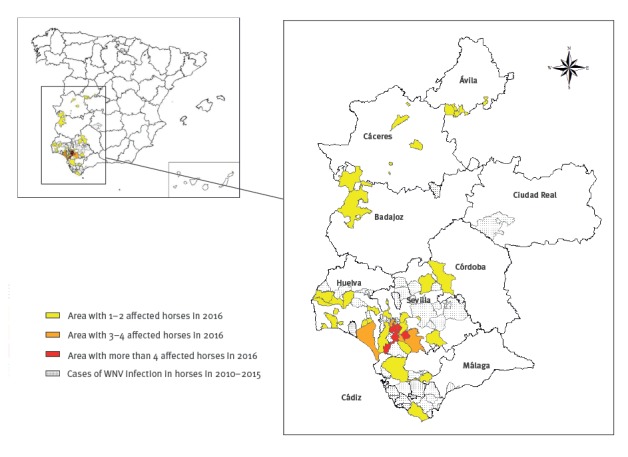
Isolation of West Nile virus in horses by district, west Andalusia, Spain, 2016 (n = 68)

**Figure 3:**
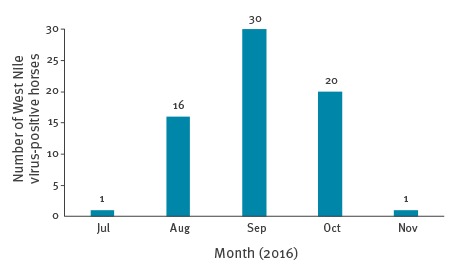
Epidemic curve of cases of West Nile virus infections in horses, west Andalusia, Spain, 2016 (n = 68)

The surveillance in horses reported 68 cases infected with WNV lineage 1 at the end of the 2016 season, in four provinces of Andalusia (Seville, Cádiz, Huelva and Cordoba; Figure 2). The first case was diagnosed on 29 July in Cádiz province, and the last in Huelva province on 17 November 2016, (Figure 3). A risk area was designated after detection of horses and/or human confirmed cases. Of the 68 horses, 75% (n=51) were detected in Seville province and 21% (n=13) in Huelva province. Almost half of the horses (n=30) were diagnosed in September.

WNV: West Nile virus.

Source: Ministry of Agriculture and Fisheries, Food and Environment of Spain (MAPAMA) and Equality on the national network for epidemiological surveillance (RENAVE).

The results in bird surveillance are not available for 2016. On October 2015 was detected WNV-lineage 1 in two partridges in Malaga [24]. *Culex pipiens* and *Culex theileri* were found in west Andalusia (Cádiz, Seville and Huelva) with last update in April 2016 [24].

## Discussion and conclusions

During 2016 WNV season, control measures were established and three WNV human cases were diagnosed. The first case, a French tourist visiting Andalusia, was communicated through the EWRS. The other two cases were diagnosed in Andalusia. Recovery was complete in all three confirmed cases. In Europe, in the 2016 WNV season, the first case in humans was reported on 20 June from Israel. There were 225 human cases reported in the EU, including 93 in Romania, 76 in Italy, 44 in Hungary, 5 in Austria, 3 in Spain, 2 in Bulgaria, 1 in Cyprus and 1 in Croatia [25].

The response in Andalusia was in accordance with the SVEA WNV protocol, first establishing risk areas and then promoting individual measures, avoiding mosquito bites and cleaning potential mosquito breeding sites (e.g. stagnant water). The summer action plan incorporated an intensive press campaign. This is one of the most important measures and countries with an endemic situation implement it every season [26]. Additionally, surveillance of unexplained meningitis and meningoencephalitis cases, which are the most frequent clinical presentations, was conducted.

The protocol describes the recommendations for tissue-sample handling and post-mortem examination and banning individuals who have been diagnosed with or have shown symptoms of WNV infection from donating blood for 120 days. All the WNV NAT screening had negative results. There is no scientific consensus on whether it is a cost-effective technique [27]. In non-risk areas, control measures in blood donations were also performed. Donors who had been in a risk area in the previous 28 days were excluded from donating blood, in line with EU legislation [26]. There were no problems with blood stocks; blood services in Andalusia work as a network and distribute blood according to need throughout the territory.

The measures taken by us are implemented in the majority of the European countries with WNV cases. For example, in Italy, where WNV is endemic, local health authorities implement an active surveillance of at-risk population and a passive surveillance of human neurological cases. In addition, Italy has a web-based national animal disease notification system. In Greece, the response is focused on awareness campaigns directed towards physicians, active surveillance and support of laboratory confirmation. There is a legal framework regarding WNV surveillance at EU level [28].

As for the microbiological results, all three cases were confirmed by WNV IgM in the CSF, which is one of the laboratory criteria for diagnosis of a WNV case [18], in line with EU case definition. Low-level viruria was demonstrated in the convalescent phase of the disease from the second case; this could be the reason why the WNV lineage by sequencing analysis of flaviviral PCR amplicons was not successful.

Investigation of WNV in urine is not currently included in the laboratory criteria to define a WNV case by the European Centre for Disease Prevention and Control [18]. Previous reports have demonstrated that viruria is associated with WNV fever and neuroinvasive infection and that higher and prolonged levels of WNV can be observed in urine vs blood samples [29,30]. The United States Centers for Disease Control and Prevention’s Guidelines for Surveillance, Prevention, and Control of WNV include virus isolation, antigen or RNA detection in body fluids other than CSF and/or serum as diagnostic criteria to confirm WNND [31]. In this study, WNV RNA could only be demonstrated in the urine from one patient with no detectable viraemia. An earlier urine sampling might have helped to improve RNA detection and genetic characterisation of WNV neuroinvasive disease.

Due to the heterogeneity of the epidemiological situation of WNV in Europe, surveillance strategies should be tailored to the unique epidemiological demands of each country to optimise efficiency and rational use of resources [26]. Countries with yearly reported outbreaks in humans or animals, like Spain, Italy or Greece, aim for early detection of WNV circulation and surveillance measures in these areas may involve active surveillance of autochthonous case in humans or animals. In these cases, active vector surveillance may not be cost-effective and human surveillance should be focused on preparedness.

Early detection and description of human cases in areas where virus circulation has been previously identified is essential for establishing prevention and control measures. A multidisciplinary approach involving trained and well-informed physicians, veterinarians and other allied professionals is necessary. Likewise, it is also fundamental to identify the affected territory in order to adopt the appropriate control measures. This allows early detection of cases and prompt escalation of appropriate control measures that are implemented at the beginning of the vector season. In Europe, WNV surveillance protocols change in accordance with the peculiarities of the WNV epidemiological situation in each country. Currently there is no WNV vaccine for humans; therefore, surveillance and control measures play a key role in the management of the spread of the disease.

As a result of this outbreak and the gathered experience, SVEA WNV protocol was updated in October 2016. Surveillance is a powerful instrument to guide targeted control measures. SVEA will continue to establish measures to control WNV disease in humans and work in partnership with the different agencies involved.
